# Allelopathy and Allelochemicals of *Leucaena* *leucocephala* as an Invasive Plant Species

**DOI:** 10.3390/plants11131672

**Published:** 2022-06-24

**Authors:** Hisashi Kato-Noguchi, Denny Kurniadie

**Affiliations:** 1 Department of Applied Biological Science, Faculty of Agriculture, Kagawa University, Miki 761-0795, Japan; 2 Department of Agronomy, Faculty of Agriculture, Universitas Padjadjaran, Jl. Raya, Bandung Sumedang Km 21, Jatinangor, Sumedang 45363, Indonesia; denny.kurniadie@unpad.ac.id

**Keywords:** allelochemical, decomposition, exudation, invasive plant, mimosine, phytotoxicity, rhizosphere soil

## Abstract

*Leucaena leucocephala* (Lam.) de Wit is native to southern Mexico and Central America and is now naturalized in more than 130 countries. The spread of *L. leucocephala* is probably due to its multipurpose use such as fodder, timber, paper pulp, shade trees, and soil amendment. However, the species is listed in the world’s 100 worst invasive alien species, and an aggressive colonizer. It forms dense monospecific stands and threatens native plant communities, especially in oceanic islands. Phytotoxic chemical interactions such as allelopathy have been reported to play an important role in the invasion of several invasive plant species. Possible evidence for allelopathy of *L. leucocephala* has also been accumulated in the literature over 30 years. The extracts, leachates, root exudates, litter, decomposing residues, and rhizosphere soil of *L. leucocephala* increased the mortality and suppressed the germination and growth of several plant species, including weeds and woody plants. Those observations suggest that *L. leucocephala* is allelopathic and contains certain allelochemicals. Those allelochemicals may release into the rhizosphere soil during decomposition process of the plant residues and root exudation. Several putative allelochemicals such as phenolic acids, flavonoids, and mimosine were identified in *L. leucocephala.* The species produces a large amount of mimosine and accumulates it in almost all parts of the plants, including leaves, stems, seeds, flowers, roots, and root nodules. The concentrations of mimosine in these parts were 0.11 to 6.4% of their dry weight. Mimosine showed growth inhibitory activity against several plant species, including some woody plants and invasive plants. Mimosine blocked cell division of protoplasts from *Petunia hybrida* hort. ex E. Vilm. between G_1_ and S phases, and disturbed the enzyme activity such as peroxidase, catalase, and IAA oxidase. Some of those identified compounds in *L. leucocephala* may be involved in its allelopathy. Therefore, the allelopathic property of *L. leucocephala* may support its invasive potential and formation of dense monospecific stands. However, the concentrations of mimosine, phenolic acids, and flavonoids in the vicinity of *L. leucocephala*, including its rhizosphere soil, have not yet been reported.

## 1. Introduction

**Figure 1:**
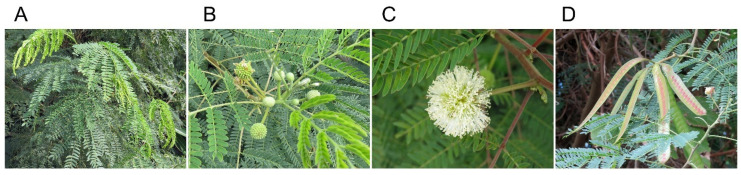
*Leucaena leucocephala*. (**A**) Leaf, (**B**) Flower head, (**C**) Flower, (**D**) Pod. Photos were taken in Yoron Island, Japan by Kato-Noguchi.

*Leucaena leucocephala* (Lam.) de Wit, belonging to Fabaceae, is native to southern Mexico and Central America [1,2]. The species is essentially a tropical species with poor cold tolerance, situated at a latitude between 30 degrees north and south of the equator. It grows well where an annual precipitation is between 650 mm and 3000 mm with dry seasons up to 4–6 months, and an average annual temperature is between 25 °C and 30 °C [3,4,5]. Three subspecies of *L. leucocephala* are recognized; ssp. *leucocephala* is shrubby and highly branched up to 5 m in height, ssp. *glabrate* is a large trunk with poorly branched up to 20 m in height, and ssp. *ixtahuacana* is medium-sized and grows up to 10 m in height with many branches [1,2,6]. The species has alternate and bipinnate leaves with 4–9 pairs of pinnae per leaf, and 13–21 pairs of leaflets per pinnae (Figure 1). The leaves show nyctinasty in the evening [7]. It is an evergreen but facultatively deciduous species under stress conditions such as low temperature and severe dryness [1,2]. *L. leucocephala* is a fast-growing species, capable of reaching reproductive maturity within 12 months, and 4 months under ideal condition [8,9]. Flower heads actively grow young shoots of 12–21 diameter, and bear 100–180 flowers per head. One flower head generates 5–20 pods. Pods are 11–19 cm long 15–21 mm wide and contain 8–18 seeds per pod (Figure 1). The species flowers all year-round and produces abundant seeds, mostly by self-fertility [2,6].

*L. leucocephala* was carried from Central America, probably as livestock fodder, to the Philippines, Guam, and West-Pacific islands between the 16th and 18th century [3,10]. The species was also introduced to plantations in Indonesia, Papua New Guinea, Malaysia, and other counties of Southeast Asia. It was then taken to Hawaii, Australia, India, East West Africa, and Caribbean islands during the 19th century [3,11]. The distribution of the species has already expanded throughout the tropic and subtropics [1,2].

One of the reasons of the spread of *L. leucocephala* is probably due to its beneficial traits. The species is recognized as a high quality and nourishing fodder tress in the tropics and subtropics. It is used for feedstuff for ruminants (cattle, water buffalos, and goats) and non-ruminants (rabbit, chickens, and fishes) [3,12]. The leaves of the species contain high levels of protein (22–28% of the dry weight) with essential amino acids such as phenylalanine, leucine, isoleucine, and histidine, and several minerals such as calcium and phosphorus [13,14,15]. It contains carbohydrates up to 20% of the dry weight [14]. The species is also used for paper pulp and timber. The wood is strong and medium density, and suitable for carpentry materials. Its caloric value as fuelwood is about 4600 calories per kg, and its biochar improves soil property of crop fields [16,17,18,19]. 

*L. leucocephala* works as shade trees in several plantations such as coffee, tea, and cacao. It also acts as a shelterbelt for a variety of crops [20,21,22]. The species is a possible candidate for the restoring vegetation covering slops, watersheds, and degraded lands to reduce erosion, and to recover vegetation [23,24,25]. In addition, its leaves and young pods are used as vegetables by local people in Central America and South Asia. Brown, red, and black dyes are extracted from its pods, leaves, and bark in Mexico. The roots and bark of the species are also used for a folk remedy, and the roots are used to get an abortion [3,26]. Thus, *L. leucocephala* is widely recognized as a multipurpose plant species.

However, despite the economic values of *L. leucocephala*, the species is listed in the 100 of the world’s worst invasive alien species [1,2]. The species was reported to be an aggressive colonizer, to form dense monospecific stands, and threaten native plant communities, especially in oceanic islands [8,27]. It has been suggested that allelopathy of *L. leucocephala* may contribute to its high invasive potential [28,29]. Allelopathy is the chemical interaction of one plant on another neighboring plants through the releasing certain secondary metabolites defined as allelochemicals, which affect the germination, growth, and establishment of the neighboring plants [30]. Possible evidence for the allelopathy of *L. leucocephala* has accumulated since the observation of its allelopathy by Chou and Kuo [28]. However, there has been no review article focusing on the allelopathy of the species. The objective of this review is to discuss possible involvement of allelopathy in the invasive potential of *L. leucocephala.* The paper provides an overview of the allelopathy and allelochemicals in the species for the discussion of its invasive characteristic. 

## 2. Field Observation

**Figure 2:**
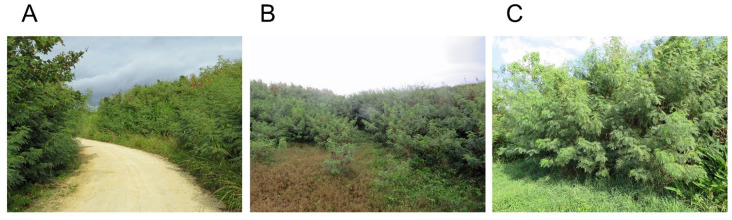
The infestation of *L. leucocephala* in Yoron Island. (**A**) Roadside, (**B**) Deserted land, (**C**) Abandoned agricultural field.

*L. leucocephala* has already naturalized in more than 130 countries in the Pacific Ocean region, Asia, South and Central America, Caribbean, Africa, Middle East, Australia, and Europe, and the number of naturalized countries is still increasing [1,2]. Ecosystems in islands are very vulnerable against invasive plant species like *L. leucocephala,* and *L. leucocephala* has caused serious problems in native plant communities in tropical and subtropical oceanic islands [27,31,32,33,34,35,36]. *L. leucocephala* is recognized as being a weed of roadsides, hillsides, forest margins, riparian habitats, deserted lands, and abandoned lands (Figure 2). The species also could invade into disturbed forests, according to the observations by Chen et al. [37] and Wolfe and Bloem [38]. 

*L. leucocephala* flowers and produces seeds all year round after reaching a reproductive stage [8,9]. Most seeds drop and stay in the proximity under the canopy of the parents, and some seeds are carried to new areas by water and the activity of animals and humans [8]. The life span of the species is relatively long (>30 years) and it produces a large number of seeds. These seeds are able to germinate after 10–20 years in good seedbank conditions, and after 1–5 years in hot and humid conditions [11,39]. Once *L. leucocephala* invades and establishes, the longevity of the plants and seedbanks may keep *L. leucocephala* stands for long time. Some seeds even germinated and established plants from the seedbanks several years after the removal of *L. leucocephala* trees [28]. 

It was hypothesized that the native species in Hawaii lowlands could maintain dominance against *L. leucocephala*, and even expand into surrounding forests dominated by *L. leucocephala* over a long time [40,41]. However, a transition back to the native forest composition has not been observed, even in 60 year old *L. leucocephala* stands, and *L. leucocephala* kept the dominance [42,43]. In addition, the observation between 1970 and 2016 in Hawaii lowlands showed a reduction in the dominance of native species in the lowland forests, and an increase in the population of non-native species, including *L. leucocephala*, in the forests [36]. The infested areas of *L. leucocephala* in the Taiwan main island increased year by year, and its annual average dispersion speed was about 3.4 ha per year [37]. *L. leucocephala* also interfered with the replacement of the native woody species in its dominant forests in Bonin Islands [31,44]. The lack of the replacement of the native plant species in *L. leucocephala* dominant forests was thought to be due to the prevention of the regeneration processes of native species by *L. leucocephala* [31]. 

*L. leucocephala* threatens native vegetations and biodiversity in the invaded areas [11,27,34]. The species richness in *L. leucocephala* invaded areas was lower than that in its uninvaded areas, and the establishment of the native plant species was hardly observed in the *L. leucocephala* invaded areas [31,34,45,46]. Seedling establishment of native plant species in *L. leucocephala* plantations was also less [45]. Those observations indicate that *L. leucocephala* reduces the biodiversity in *L. leucocephala* invaded areas because of the suppression of the germination and establishment of other plant species, including native plant species.

Sunlight intensity of the forest floors under the canopy of *L. leucocephala* trees was reported to be sufficient for the growth of understory plants [28]. There was also not much difference in the canopy openness and light conditions on the forest floors between *L. leucocephala* dominant forests and others [31]. In addition, most abundant species in *L. leucocephala* forests in Kaoshu, South Taiwan, were shade-intolerant plant species, *Axonopus compressus* (Sw.) P.Beauv., and *Ageratum conyzoides* L. [28], which indicates that the light intensity was enough for the shade-intolerant plant species to grow.

Legume species, *L. leucocephala*, can fix nitrogen by a symbiosis with nitrogen-fixing bacteria. Nitrogen levels in the soil under *L. leucocephala* invaded stands were high compared to outside the stands [47,48]. On the other hand, net nitrification and net mineralization in the soil under *L. leucocephala* forests were faster than those in the soil under native forests, and total nitrogen and carbon levels in the soil under *L. leucocephala* forests were much less than those in the soil under native forests [33]. High nitrification activity in the soil under *L. leucocephala* forests may cause excessive nitrogen losses through the leaching from the soil due to high mobility of nitrate [49]. In addition, other conditions such as soil moisture and other soil conditions were comparable between *L. leucocephala* invaded forests and non-invaded forests [28]. 

Although *L. leucocephala* was observed to suppresses the regeneration and establishment processes of native plant species, the difference in light and other conditions between *L. leucocephala* invaded forests and non-invaded forests was not apparent. There has also been no clear explanation for the suppression of *L. leucocephala* on native plant species. However, it was observed that the survival and growth of a native woody plant species, *Erythrina velutina* Willd., was suppressed by the presence of *L. leucocephala* in the field experiments under relatively controlled conditions [34]. The experiments under controlled conditions may illuminate the possible involvement of light and other environmental factors and suggest the involvement of other factors, such as allelopathy, in the suppression. Thus, allelopathy of the species seems to contribute to the suppression of the regeneration and establishment processes of native plant species [28,29].

## 3. Allelopathy

Allelopathy is chemical interaction among plants and caused by allelochemicals [30], which are produced in plants and released into the vicinity of the plants including rhizosphere soil either by root exudation, decomposition of plant litter and residues, and rainfall leachates and volatilization from the plant parts [50,51,52]. The allelopathic potential of plant extracts, leachates, litter, residues, and rhizosphere soil of *L. leucocephala* was evaluated over 30 years (Table 1). Those observations suggest that *L. leucocephala* may produce and accumulate certain allelochemicals and release them into the neighboring environments either by rainfall leachates, decomposition process of plant parts, and root exudation.

### 3.1. Plant Extract

Allelopathic activity of the extracts of leaves, seeds, bark, and aerial parts of *L. leucocephala* on crops and weeds were determined since allelochemicals are synthesized and accumulated in certain plant parts [30,50,51,52]. Aqueous extracts of the leaves of *L. leucocephala* suppressed the radicle growth of *Lactuca sativa* L. and *Oryza sativa* L. seedlings [28], and the seedling growth of *Ischaemum rugosum* Saisb and *Vigna radiata* (L.) R. Wilczek [53]. Its aqueous leaf and seed extracts showed the inhibitory activity on the germination and seedling growth of three weed species, *Ageratum conyzoides* L. *Tridax procumbens* L., and *Emilia sonchifolia* (L.) DC. Ex Wight [54]. Aqueous leaf, seed, and bark extracts of *L. leucocephala* inhibited the germination, growth, and crop yield of *Zea mays* L. under pot culture conditions [55]. It was also reported that aqueous extracts of the aerial part of *L. leucocephala* suppressed the growth of two weeds, *Bidens pilosa* L. and *Amaranthus hybridus* L., under laboratory and greenhouse conditions [56]. The extracts showed the inhibition of the growth of a weed, *Ipomoea grandifolia* (Dammer) O′Donell, and the inhibition of the germination and growth of two weeds, *Arrowleaf sida* L. and *Bidens pilosa* L. [57].

The inhibitory mechanism of the extracts of *L. leucocephala* on the plant growth was also investigated. Aqueous extracts of its aerial parts inhibited the cell division of *Zea mays* L. roots, and increased peroxidase activity in the roots [58]. Aqueous leaf extracts of *L. leucocephala* disturbed the cell division of the radicles of *Pisum sativum* L. [59]. Those observations indicate that the extracts of leaves, seeds, bark, and aerial parts of *L. leucocephala* possess inhibitory activity on the germination and growth of several plant species, and probably contain water extractable allelochemicals, which may disturb cell division and affect some enzyme activities. 

### 3.2. Leachate

For the simulation of rainfall conditions, plant tissues were soaked in water, and its supernatant was used as leaches from the tissues by rainfall [60,61,62,63,64]. The senescent leaves of *L. leucocephala* was soaked in water for 48 h, and its supernatant showed inhibitory activity on the germination and growth of *Raphanus sativus* L. [62]. The soaking water of *L. leucocephala* leaves also enhanced electrolyte leakage from the leaf cells of *Eichhornia crassipes* (Martius) Solms. and increased the activities of catalase and ascorbate peroxidase in the leaves [63]. Those observations suggest that the leaches may contain certain allelochemicals, which may cause growth inhibition and affect cell membrane permeability and enzyme activities. They also imply that certain allelochemicals would be possible to be released from the leaves of *L. leucocephala* into the neighboring environments as rainfall leachates. 

### 3.3. Plant Litter and Residue

Leaf litter of *L. leucocephala* was mixed with soil, and the seeds of a woody plant, *Albiza procera* (Roxb.) Benth., and crop plants, *Vigna unguiculata* (L.) Walp., *Cicer arietinum* L. and *Cajanus cajan* (L.) Millsp. were sown into the mixture. The treatments resulted in the suppression of the germination and growth of these test plant species [64]. Soil mixture with decomposing leaves of *L. leucocephala* increased the mortality of five tree species, *Alnus formosana* (Burkill) Makino, *Acacia confusa* Marr., *Liquidambar formosana* Hance, *Casuarina glauca* Sieber, and *Mimosa pudica* L. [28].

Aqueous extracts of *L. leucocephala* litter, which accumulated on the forest floors, showed the suppression of the germination and radicle growth of *Lolium multiflorum* Lam. [28], and *Ageratum conyzoides* L. *Tridax procumbens* L., and *Emilia sonchifolia* (L.) DC. ex Wight [65]. Leaf mulch of *L. leucocephala* covered on soil surface or mixed with soil inhibited the germination and growth of *Vigna unguiculata* (L.) Walp., and the root nodulation of *V. unguiculate* [66]. Those observations indicate that leaf litter and residues of *L. leucocephala* may contain certain allelochemicals, and some of them may be liberated into the soil during their decomposition processes.

### 3.4. Rhizosphere Soil and Root Exudate

The seeds of *Ageratum conyzoides* L., *Tridax procumbens* L., and *Emilia sonchifolia* (L.) DC. ex Wight were sown into the soil collected from *L. leucocephala* infested areas. The treatments resulted in the suppression of the germination and growth of those plant species [65]. Rhizosphere soil of *L. leucocephala* also inhibited the germination and growth of *Vigna radiata* (L.) R.Wilczek and *Glycine max* L. [67]. Aqueous extracts of the soil of the forest floors under *L. leucocephala* trees showed the inhibition of the radicle growth of *Lactuca sativa* L. [28]. In addition, root exudates from *L. leucocephala* showed the suppression of the germination and growth of *Ageratum conyzoides* L., *Tridax procumbens* L., and *Emilia sonchifolia* (L.) DC. ex Wight [65]. Those observations suggest that rhizosphere soil of *L. leucocephala* may contain certain allelochemicals, which may be supplied through root exudation, decomposition of plant litter and residues, and rainfall leachates.

## 4. Allelochemical

**Figure 3:**
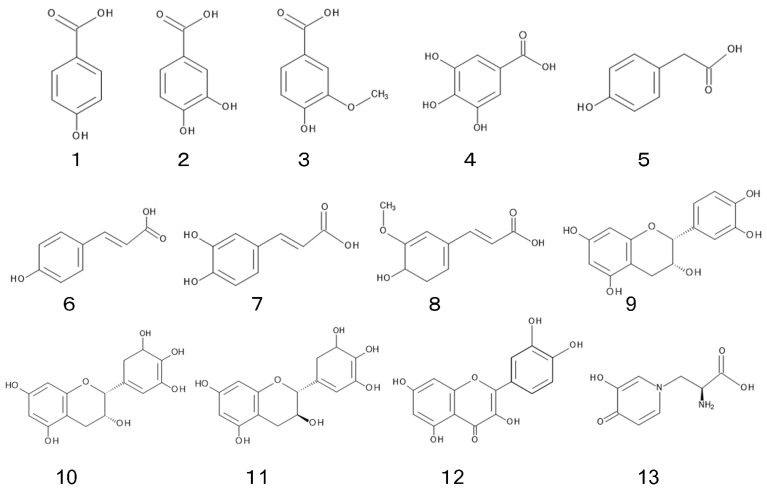
Allelochemicals identified in *L. leucocephala*. (**1**) *p*-hydroxybenzoic acid, (**2**) protocatechuic acid, (**3**) vanillic acid, (**4**) gallic acid, (**5**) *p*-hydroxyphenylacetic acid, (**6**) *p*-hydroxycinnamic acid, (**7**) caffeic acid, (**8**) ferulic acid, **(9**) epicatechin, (**10**) epigallocatechin, (**11**) gallocatechin, (**12**) quercetin, (**13**) mimosine.

Phenolic acids, flavonoids, and mimosine were isolated and identified from *L. leucocephala* as its allelopathic agents (Figure 3).

### 4.1. Phenolic Acid 

Phenolic acids such as *p*-hydroxybenzoic acid (**1**), protocatechuic acid (**2**), vanillic acid (**3**), gallic acid (**4**), *p*-hydroxyphenylacetic acid (**5**), *p*-hydroxycinnamic acid (**6**), caffeic acid (**7**), and ferulic acid (**8**) were identified in the leaves of *L. leucocephala*. Total concentrations of those phenolic acids in young leaves were 2-fold greater than those in mature leaves [28]. The concentration of total phenolic compounds in *L. leucocephala* plants was estimated to be 1.3 to 2.8 mg g^−1^ of dry weight of the plants [68]. Phenolic acids have been found in a wide range of plants, plant residues, and soils, and their involvement has been often mentioned in the allelopathy of those plant species [69,70]. 

The main allelochemicals found in the rhizosphere soil of *Ageratum conyzoides* L., which is known as an invasive plant species, were gallic acid, *p*-hydroxybenzoic acid, ferulic acid, and *p*-coumaric acid [71]. The concentration of total phenolic acids in the rhizosphere soil of *Lantana camara* L., which is also known as an invasive plant, was 27.6% higher than that in the soil of *L. camara* un-infested areas [72]. The inhibitory activity of phenolic acids on the plant growth and germination was reported to be concentration-dependent. Phenolic acids affect cell membrane permeability, inhibit cell division, and interfere with several enzyme activities and major physiological processes, such as nutrient uptake, water balance and stomatal functions, phytohormone synthesis, protein synthesis, respiration, and metabolism of some other secondary metabolites [69,73,74]. However, only limited information is available on phenolic acids in *L. leucocephala* plants and its rhizosphere soils.

### 4.2. Flavonoid

Flavonoids such as epicatechin (**9**), epigallocatechin (**10**), and gallocatechin (**11**) were identified in *L. leucocephala* roots. Those compounds inhibited the nitrification process, which is an important step in the nitrogen cycle in soil [75]. Quercetin (**12**) and other 16 flavonoids were identified in *L. leucocephala* leaves, and some of them showed antioxidant activity [76]. *L. leucocephala* was reported to contain condensed tannins, which may contribute towards the plant resistance to pathogens and insects. [77,78,79]. 

Epicatechin showed the growth inhibitory activity on several plant species [80,81]. Catechin, diastereomer of epicatechin, also has potent growth inhibitory activity, and was once considered to be involved in the invasion of *Centaurea stoebe* L. into North America. In their novel weapon hypothesis, the invasive plant species, *C. stoebe* would release certain amounts of catechin into the rhizosphere soil, and the released catechin suppresses the regeneration process of the native plant species through the inhibition of their germination and growth [82,83]. However, the actual catechin levels in the soil were very low and could not cause significant growth inhibition of native plant species [84]. There has also been no information about the concentration of the flavonoids in the rhizosphere soil of *L. leucocephala*.

### 4.3. Mimosine

Mimosine (**13**); L-mimosine, synonym; leucenol) is a non-protein amino acid. It was first isolated from *Mimosa pudica* L [85], and found in some other species of the genus, *Mimosa* and *Leucaena*, including *L. leucocephala* [86,87,88]. Mimosine possesses a wide range of pharmacological and biological properties, such as anti-tumor, apoptotic, anti-inflammation, anti-viral, and cell cycle blocking effects [89]. It also possesses the inhibitory activity on the germination and growth of several plant species [28,90,91]. Therefore, mimosine is possibly involved in the allelopathy of *L. leucocephala*. 

**Figure 4:**
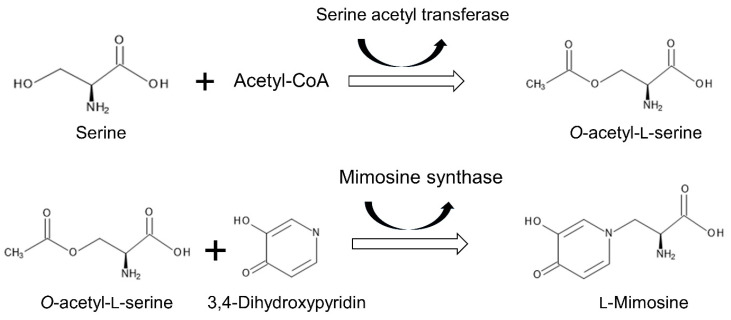
Pathway of mimosine biosynthesis.

Mimosine is synthesized by a reaction comparable to the pathway of cysteine biosynthesis. Both reactions use *O*-acetyl-L-serine as a donor of the alanyl group. Serine acetyl transferase [2.5.1.30] catalyzes a reaction to form *O*-acetyl-L-serine from serine and acetyl-CoA. Mimosine synthase [2.5.1.52] catalyzes a reaction of L-mimosine formation from *O*-acetyl-L-serine and 3,4-dihydroxypyridin (Figure 4) [92,93,94,95].

*L. leucocephala* produces a large amount of mimosine and accumulates mimosine in almost all parts of the plants, including leaves, stems, seeds, flowers, roots, and root nodules [96,97]. The concentrations of mimosine in these parts were 0.11% to 6.4% of their dry weight. Younger leaves contained mimosine greater than senescent leaves [97,98]. The concentration of mimosine in growing shoot tips was 22.2% of its dry weight [98]. UV-C radiation, jasmonic acid, and ethephone, which is an ethylene-releasing compound, stimulated mimosine accumulation in shoots and roots of *L. leucocephala* [99]. Salicylic acid and mechanical damage, and NaCl treatments also increased the mimosine concentration [98,100]. Jasmonic acid, ethylene, and salicylic acid act as environmental stress signaling molecules, and are involved in the stress-related gene expression [100,101,102,103]. Those observations suggest that environmental and ontogenesis factors may affect mimosine biosynthesis and accumulation in *L. leucocephala*.

Mimosine at 100 ppm showed the root growth inhibition on *Brassica rapa* L., *Phaseolus vulgaris* L., *Bidens pilosa* L., *Lolium multiflorum* Lam., and *Mimosa pudica* L. by 40–95%, and their shoot growth inhibition by 11–93% [97]. Mimosine at approximately 100 ppm also inhibited the shoot and root growth of *Sesbania herbacea* (Mill.) McVaugh, *Senna obtusifolia* (L.) H.S.Irwin et Barneby [104], and *Ageratum conyzoides* L., *Emilia sonchifolia* (L.) DC. ex Wight and *Tridax procumbens* L. [105]. In addition, the seedlings of *Ageratum conyzoides* (L.) L. were killed 7 days after a mimosine treatment at 50 ppm [28]. Those observations indicate that momosine has inhibitory activity against weeds such as *Bidens pilosa*, *Lolium multiflorum*, *Ageratum conyzoides*, *Senna obtusifolia, Emilia sonchifolia*, and *Tridax procumbens,* and shrubs such as *Sesbania herbacea* and *Mimosa pudica*. The shrub and weed species may have an opportunity to compete with *L. leucocephala* in natural conditions for resources such as water, light, and nutrition, and many of them are also listed as invasive plant species. Mimosine may enhance the competitive ability of *L. leucocephala* against those competitors because of its growth inhibitory effects.

On the other hand, mimosine has a weak inhibitory effect on *L. leucocephala* itself. The inhibition of mimosine at 100 ppm on the root and shoot growth of *L. leucocephala* was only by 2.4% and 8.7%, respectively [28,97]. A mimosine degradation enzyme, minosinase was found in *L. leucocephala*, and its gene expression was also high [106,107]. Therefore, weak inhibitory activity of mimosine on *L. leucocephala* may be due to the existence of the degradation enzyme. 

Mimosine was reported to suppress the cell cycle of protoplasts from *Petunia hybrida* hort. ex E.Vilm. between G_1_ and S phases [106] (Perennes et al., 1993), which is consistent with the inhibition of cell division of *Pisum sativum* radicles and *Zea mays* roots by the aqueous extracts of *L. leucocephala* [58,59], as described in the previous section. It was also reported to block cell division in mammalian and insect cells, blocking entry into S phase from late G_1_ phase and suppressing elongation of DNA replication (S phase) [89,108,109,110,111]. The activities of nitrate reductase, peroxidase, catalase, and IAA oxidase were inhibited by mimosine treatments in *Oryza sativa* L. seedling [112]. Root growth inhibition of *Glycine max* (L.) Merr. by mimosine treatments was correlated with the reduction in the activities of phenylalanine ammonia-lyase and peroxidase [113]. Those observations suggest that the inhibitory effects of mimosine may be caused by the disturbance of some enzyme functions and cell division.

Mimosine was also reported to retard the growth and development of the larvae of a harmful cosmopolitan insect *Tribolium castaneum* Herbst [114]. Aqueous extracts of *L. leucocephala* increased the mortality of the nymphs of *Bemisia tabaci* Gennadius [115]. Mimosine and the extracts of *L. leucocephala* also increased the mortality of a nematode, *Meloidogyne incognita* Kofoid and White [116,117]. High defense capacity of the plants against natural enemies such as herbivores, nematodes, and pathogens was thought to be essential for the plant survival and increasing population [118,119,120]. Mimosine may contribute to the survival and increasing population of *L. leucocephala* against the attacks of herbivores and nematodes. 

It was reported that *L. leucocephala* secreted mimosine at 1–5 μg g^−1^ dry weight plant per day into growth medium [96]. *L. leucocephala* contains a large amount of mimosine in all parts of plants as described above, and some amounts of mimosine may be liberated into its rhizosphere soil during the decomposition process of the plant litter and residues and may work as an allelopathic agent. However, there has been no information on the levels of mimosine in the vicinity of *L. leucocephala*, including its rhizosphere soil. The information is necessary to evaluate mimosine contribution to the allelopathy of *L. leucocephala*.

Large number of secondary metabolites in many chemical classes, such as triterpenes, tannins, saponins, steroids, glyceride, and benzenoids, have been isolated and identified in the seeds, leaves, stems, and roots in *L. leucocephala* [121,122,123,124,125,126]. Some of those compounds have been associated with pharmacological properties. Many of the secondary metabolites from the invasive plants have been reported to show multiple effects such as allelopathic, anti-herbivore, anti-fungal, and anti-microbial activity. Those compounds are able to increase the fitness of the plants in invasive ranges [120,127,128]. Therefore, some of those compounds found in *L. leucocephala* may also work as allelopathic agents and enhance the competitive ability of *L. leucocephala* against neighboring plant species.

## 5. Conclusions

Although the economic value of *L. leucocephala* is widely recognized, the species is listed in the world’s 100 worst invasive alien species. It is an aggressive colonizer and forms dense monospecific stands. It interferes the regeneration and replacement of native plant species in its dominant forests. The species richness in *L. leucocephala* invaded forests was lower than that in its uninvaded forests, and seedling establishment of native plant species under *L. leucocephala* invaded areas was also low. Sunlight intensity and other conditions of the forest floors between *L. leucocephala* forests and native forests were not apparent. In addition, plant extracts, leachates, root exudates, plant litter and residues, and rhizosphere soil of *L. leucocephala* showed the enhancement of the mortality and suppression of the germination and growth of several plant species including weeds and woody plants. Those observations suggest that *L. leucocephala* is allelopathic and contains allelochemicals which affect the plant mortality, germination, and growth, and some of the allelochemicals may be released into the vicinity of *L. leucocephala*, including its rhizosphere soil.

*L. leucocephala* produces a large amount of mimosine and accumulates it in almost all parts of the plants. Mimosine showed growth inhibitory activity against several plant species including some shrubs and another invasive plant species. Mimosine blocked cell division of protoplasts from *Petunia hybrida* between G_1_ and S phases, and disturbed some enzyme activities such as peroxidase, catalase, and IAA oxidase. In addition, several phenolic acids and flavonoids were identified in *L. leucocephala*. However, the concentrations of mimosine, phenolic acids, and flavonoids in the rhizosphere soil and vicinity of *L. leucocephala* have not yet been reported. The information is essential to evaluate the contribution of mimosine, phenolic acids, and flavonoids to the allelopathy of *L. leucocephala*.

## Figures and Tables

**Table 1:** Allelopathic activities of plant extracts, leachates, litter, residues, rhizosphere soil, and root exudates of *L. leucocephala*.

Source	Target Plant Species	Inhibition	Stimulation	Condition	Reference
**Plant extract**					
Leaf	*Lactuca sativa, Oryza sativa*	Growth		Laboratory	[28]
	*Ischaemum rugosum, Vigna radiata*	Growth		Laboratory	[53]
Leaf, seed	*procumbens, Emilia sonchifolia*	Germination, growth		Laboratory	[54]
Leaf, bark, seed	*Zea mays*	Germination, growth, crop yield		Laboratory, greenhouse	[55]
Aerial part	*Bidens pilosa, Amaranthus hybridus*	Growth		Laboratory, greenhouse	[56]
	*Ipomoea grandifolia, Arrowleaf sida, Bidens pilosa*	Growth		Laboratory	[57]
Aerial part	*Zea mays*	Cell division	Peroxidase activity	Laboratory	[58]
Leaf	*Pisum sativum*	Cell division		Laboratory	[59]
	*Ageratum conyzoides, Tridax*				
**Leachate**					
Senescent leaf	*Raphanus sativus*	Germination, growth		Laboratory	[60,61,62]
Leaf	*Eichhornia crassipes*		Electrolyte leakage, catalase, and ascorbate peroxidase activities	Laboratory	[63]
**Litter, residue**					
Leaf	*Albiza procera, Vigna unguiculata, Cicer arietinum, Cajanus cajan*	Germination, growth		Greenhouse	[64]
Decomposing leaf	*Alnus formosana, Acacia confusa, Liquidambar formosana, Casuarina glauca, Mimosa pudica*	Mortality		Greenhouse	[28]
Liter extract	*Lolium multiflorum*	Germination, growth		Laboratory	[28]
Liter extract	*Ageratum conyzoides, Tridax procumbens, Emilia sonchifolia*	Germination, growth		Laboratory	[65]
Leaf mulch	*Vigna unguiculata*	Germination, growth, nodulation		Greenhouse	[66]
**Soil**					
Rhizosphere soil	*Ageratum conyzoides, Tridax procumbens, Emilia sonchifolia*	Germination, growth		Greenhouse	[65]
	*Vigna radiata, Glycine max*	Germination, growth		Greenhouse	[67]
Soil extract	*Lactuca sativa*	Growth		Laboratory	[28]
**Root exudate**	*Ageratum conyzoides, Tridax procumbens, Emilia sonchifolia*	Germination, growth		Laboratory	[65]
